# Associations among Race/Ethnicity, ApoC-III Genotypes, and Lipids in HIV-1-Infected Individuals on Antiretroviral Therapy

**DOI:** 10.1371/journal.pmed.0030052

**Published:** 2006-01-24

**Authors:** Andrea S Foulkes, David A Wohl, Ian Frank, Elaine Puleo, Stephanie Restine, Megan L Wolfe, Michael P Dube, Pablo Tebas, Muredach P Reilly

**Affiliations:** **1**School of Public Health and Health Sciences, University of Massachusetts, Amherst, Massachusetts, United States of America; **2**University of North Carolina, Chapel Hill, North Carolina, United States of America; **3**School of Medicine, University of Pennsylvania, Philadelphia, Pennsylvania, United States of America; **4**Indiana University, Indianapolis, Indiana, United States of America; University of AmsterdamNetherlands

## Abstract

**Background:**

Protease inhibitors (PIs) are associated with hypertriglyceridemia and atherogenic dyslipidemia. Identifying HIV-1-infected individuals who are at increased risk of PI-related dyslipidemia will facilitate therapeutic choices that maintain viral suppression while reducing risk of atherosclerotic diseases. Apolipoprotein C-III (apoC-III) gene variants, which vary by race/ethnicity, have been associated with a lipid profile that resembles PI-induced dyslipidemia. However, the association of race/ethnicity, or candidate gene effects across race/ethnicity, with plasma lipid levels in HIV-1-infected individuals, has not been reported.

**Methods and Findings:**

A cross-sectional analysis of race/ethnicity, apoC-III/apoA-I genotypes, and PI exposure on plasma lipids was performed in AIDS Clinical Trial Group studies (*n* = 626). Race/ethnicity was a highly significant predictor of plasma lipids in fully adjusted models. Furthermore, in stratified analyses, the effect of PI exposure appeared to differ across race/ethnicity. Black/non-Hispanic, compared with White/non-Hispanics and Hispanics, had lower plasma triglyceride (TG) levels overall, but the greatest increase in TG levels when exposed to PIs. In Hispanics, current PI antiretroviral therapy (ART) exposure was associated with a significantly smaller increase in TGs among patients with variant alleles at apoC-III-482, −455, and Intron 1, or at a composite apoC-III genotype, compared with patients with the wild-type genotypes.

**Conclusions:**

In the first pharmacogenetic study of its kind in HIV-1 disease, we found race/ethnic-specific differences in plasma lipid levels on ART, as well as differences in the influence of the apoC-III gene on the development of PI-related hypertriglyceridemia. Given the multi-ethnic distribution of HIV-1 infection, our findings underscore the need for future studies of metabolic and cardiovascular complications of ART that specifically account for racial/ethnic heterogeneity, particularly when assessing candidate gene effects.

## Abstract 2

This cross-sectional analysis of race/ethnicity, apoC-III/apoA-I genotypes, and protease inhibitor exposure on plasma lipids showed that race/ethnicity was a highly significant predictor of plasma lipids

## Introduction

The use of potent antiretroviral therapy (ART) in patients with HIV-1 is associated with a cluster of metabolic complications, including atherogenic dyslipidemia [1–3]. Analyses of data in 17,852 patients from the Data Collection on Adverse Events of Anti-HIV Drugs study document class effects of ART on lipid profiles, with dyslipidemias observed most commonly in patients receiving protease inhibitors (PIs) [4,5]. Lipid abnormalities on PIs are characterized by elevated triglycerides (TGs), low high-density lipoprotein cholesterol (HDL-c) and increased apolipoprotein (apo) B, containing very low-density lipoprotein remnants and small low-density lipoprotein particles [2,3,6,7]. Recent studies suggest that PI-based ART is associated with an increased risk for atherosclerotic cardiovascular (CV) events [4,8,9] and has raised concerns for a future epidemic of CV disease in HIV-1 patients for whom life-long ART may be required for control of viral replication. Strategies that identify HIV-1 individuals at increased risk of ART-related metabolic complications are likely to facilitate rational decision making when selecting ART regimens, as well as early use of appropriate preventive CV therapies in those at greatest risk.

The pathophysiology of dyslipidemia in ART-treated HIV patients is multi-factorial and involves drug effects on lipid metabolism [2,10], insulin signaling and adipose tissue [11], immunologic or viral factors [12], and host genetics [13–15]. Lipid abnormalities have been associated with almost all PIs, but vary with specific PIs, and are most frequent in patients taking ritonavir (RTV) or RTV-“boosted” PI combination regimens [5,16]. The direct effect of PIs on lipid metabolism is evident by elevations in TG following short courses of treatment in HIV-1-uninfected, healthy individuals [17–19]. PIs modulate both the production of apoB particles and their clearance [2,10,20]. Notably, PI ART-related dyslipidemia resembles that observed in familial combined hyperlipidemia [21], suggesting a potential role for variation in lipoprotein genes that have been linked to this relatively common inherited dyslipidemia [22].

ApoC-III is a 79-amino-acid protein whose plasma levels are directly correlated with TGs in the general population [23]. Although the in vivo function of apoC-III is poorly understood [24], in vitro studies and gene manipulation in mouse models have implicated apoC-III in regulating lipolysis of TG-rich lipoprotein [25], and in modulating remnant particle clearance by the liver [26,27]. Several studies have established a complex interaction of genetic variation within apoC-III, and the apoA-I/C-III/A-IV/AV cluster, with plasma TG levels [24,28–30]. Recently, two groups reported a marked increase in plasma TGs in HIV-1-infected patients on PI ART regimens when they also carried a combination of apoC-III and apoE gene variants [13,15], although these studies were restricted almost entirely to Caucasians.

Despite a well-described relationship of race/ethnicity with lipoproteins in the general population [31–33], there has been little consideration of ethnicity in the development of metabolic complications in HIV-1-infected individuals. Such differences may be of specific relevance in ART-associated dyslipidemia given the multi-ethnic distribution of HIV-1 infection and evidence for ethnic differences in linkage disequilibrium (LD) patterns for several lipoprotein genes [34–36]. We hypothesized that ethnic LD patterns in apoC-III [36–38], as well as distinct susceptibility/resistance alleles for lipid abnormalities [28–30,39], and prior evidence for modification of apoC-III effects by ethnicity [38,40], would result in differences across racial/ethnic stratum in the association of apoC-III and ART with plasma lipids in HIV-1 infection.

We describe results of analyses from an ongoing project designed to identify candidate genes that place ART-treated HIV-1-infected individuals at higher risk of developing dyslipidemia. As a primary hypothesis, we aimed to determine whether race/ethnicity is a significant predictor of plasma lipids and ART-associated lipid abnormalities in HIV-1-infected individuals and whether apoC-III/apoA-I gene variants interacted with PI exposure to predict increased plasma TGs within race/ethnicity strata.

## Methods

### Study Participants

As part of New Work Concept Sheet (NWCS) 224, we performed a cross-sectional analysis of 626 HIV participants enrolled in selected AIDS Clinical Trial Group (ACTG) studies (28% from A5005s, 11% from A5068, 18% from A5087, 19% from ACTG 372, and 23% from A5116) who also had consented to collection and storage of their blood for genetic studies (A5128). ACTG studies were approved by local institutional review boards at each institution where participants were recruited and all participants gave written informed consent. These specific ACTG studies were selected because appropriate data, including fasting plasma lipids and DNA samples, were available and all participants were on ART at time of sampling. Study A5005s was the metabolic sub-study of ACTG 384, a six-arm comparison of three- or four-drug ARTs with zidovudine and lamivudine, or stavudine and didanosine, combined with efavirenz (EFV), nelfinavir (NFV), or both, in ART-naïve individuals [41]. ACTG 5068 compared two strategies, treatment interruption and vaccination with ALVAC vCP1452, to augment HIV-specific immune responses in individuals on stable ART. Protocol A5087 enrolled ART-treated participants with dyslipidemia who were randomized to one of two lipid-lowering interventions**.** ACTG 372 was a rollover study for ACTG 320 to compare individuals receiving abacavir or a placebo in combination with zidovudine, lamivudine, and indinavir. Finally, A5116 was a class-sparing and regimen-simplification study for patients with advanced HIV disease. A more detailed description of the above mentioned studies can be found at http://www.clinicaltrials.gov. In our analysis, fasting lipids were recorded in all patients in A5087 prior to starting lipid-lowering therapy. Fasting lipids were recorded for patients in A5005 at least 32 wk after start of ART, and for all other studies at entry. Because lipid levels prior to HIV therapy were not consistently available, our study represents an analysis of treatment lipids across these ACTG studies.

Among study participants, *n* = 440 were on stable PI ART (*n* = 398 on a non-RTV regimen) (and *n* = 42 on a RTV regimen; median exposure 976 d; interquartile ranges [IQR] = 244, 1245) or non-PI ART (*n* = 131; 78% EFV and 13% NFV-containing regimens). Patients with short exposure to PI ART (<90 d; *n* = 13), patients not currently on PIs but who were exposed to a PI within 30 d (*n* = 2), and patients with unknown durations of exposure (*n* = 40) were excluded from analysis assessing PI effects.

### Evaluated Parameters

Study participants were evaluated through their respective ACTG protocols. Demographic and clinical information was provided to the NWCS224 team in a blinded, de-linked manner from the Statistical and Data Analysis Center of the Center for Biostatistics and AIDS Research at the Harvard School of Public Health. Race/ethnicity data were self-reported in these ACTG studies with race categorized as Black, Caucasian, American Indian, Alaska Native, Native Hawaiian, other Pacific Islander, or Asian; and ethnicity as Hispanic or non-Hispanic. For the purpose of our analysis, a single three-level race/ethnicity variable was defined, which represented 97.6% of the full sample: White/non-Hispanic (White), Black/non-Hispanic (Black), or Hispanic. Clinical information collected included medical histories, concomitant medications, ART history, CD4 counts, viral loads, height, weight, fasting plasma lipoproteins (TG, HDL-c, total cholesterol), and glucose. Fasting plasma lipids were measured according to the specific parent ACTG protocols. Non-HDL-c was calculated by subtracting HDL-c from total cholesterol. For the measured lipid data, >97% was confirmed to have been taken in the fasting state, the other <3% were presumed to be fasting based on study specific criteria. Body mass index was calculated as weight divided by height squared.

### Genotyping

DNA samples were isolated from blood (PUREGENE blood kits; Gentra Systems, Minneapolis, Minnesota, United States) at the ACTG DNA core laboratory at Vanderbilt University, Nashville, Tennessee, United States. We genotyped six single nucleotide polymorphisms (SNPs) in the apoC-III/apoA-I cluster, five in apoC-III (−482C/T [rs2854117], −455T/C [rs2854116], intron 1 (466)G/C [rs2070669], Gly34Gly C/T [rs4520], and exon 4 SstI 3238(5)C/G [rs5128]), and one in apoA-I (XmnI; −2500C/T; chromosome position 11626561, May 2004 assembly [http://www.genome.ucsc.edu]). SNPs were chosen based on prior knowledge of association with plasma lipoproteins (apoC-III-482C/T, −455T/C, SstI 3238C/G, and apoA-I-2500C/T) [24,28–30] and to provide additional information of LD across apoC-III in these analyses (intron 1 G/C and Gly34Gly in apoC-III). Genotyping was performed at Penn Genotyping Core employing Taqman (Applied Biosystems, Foster City, California, United States) ABI SNP genotyping assays were performed using the ABI 7900HT.

### Statistical Analysis

Analyses were performed using Splus for Windows, Version 6.2 and R, version 2.0. Demographic and clinical characteristics are given overall and by specific ACTG study. Data are reported as medians and IQR for continuous variables, and as proportions for categorical variables. Raw lipoprotein data are presented by race/ethnicity, gender, and drug exposures. Log transformations were employed to normalize lipoprotein data for the purpose of modeling.

As a first step, we assessed the main effects of race/ethnicity and PI exposure on plasma lipids, using multiple linear regression modeling. As a primary analysis, models were fitted treating PI exposure as a three-level factor (no current PI exposure, currently exposed to a non-RTV–containing PI regimen, and currently exposed to a RTV-containing regimen). An analysis of PI exposure as an indicator for any current PI use was also performed. Fully adjusted multivariable models included race/ethnicity, ART exposures (current use of PI, stavudine, EFV, and NFV), age, gender, study, CD4 count, and use of lipid-lowering therapy. Maximum likelihood coefficient estimates (reported as estimated fold increases in each lipid outcome on the un-logged scale), 95% confidence intervals (CI) for the true effects, and F-test statistics are reported. In addition, separate multivariable models were fitted for each SNP to assess their independent main effects on lipid outcomes. SNPs were coded as binary indicators for the presence of at least one variant allele (i.e., heterozygous or homozygous rare.) Although useful for comparison with published studies, a notable limitation of this first step is that it does not account for potential interaction between race/ethnicity, PI exposure, and genotypes.

We performed all subsequent analyses stratified by race/ethnicity in order to address our primary hypothesis of an interaction between genotype and PI exposure on TGs within racial/ethnic stratum. Maximum likelihood estimates and 95% CIs for main effects of PI exposure and interaction effects between PI exposure and apoC-III/apoA-I SNPs were estimated within race/ethnicity stratum, based on fully adjusted multivariable linear regression models. In these models, PI exposure was treated as a two-level indicator for any current exposure because there is no mechanistic data to support differential interaction effects of apoC-III genotypes with distinct PIs. Estimated fold increases in TGs and corresponding 95% CIs on the un-logged scale are again reported and significance is based on a two-sided Wald test at the 0.05 level. Predicted TG levels and 95% CIs, based on these fully adjusted multivariable models, are provided for non-PI and PI groups within racial/ethnic stratum, with and without further stratification by genotype.

Due to the high degree of concordance among the observed apoC-III SNPs, an additional composite genotype analysis is presented. As a primary analysis, the three apoC-III SNPs described by Tarr et al. (−482, −455, and SstI [3238]) [15] were considered, and a new binary variable for the presence of one or more variant allele at any number of these three SNPs was created. The interaction between this composite genotype variable and PI exposure was assessed within racial/ethnic stratum as described above for the single SNP PI interaction. Although power for three-way interactions was limited, the interaction of individual SNPs and the composite genotype, with race/ethnicity and PI exposure was estimated and tested for completeness.

Estimation of apoC-III haplotype frequencies within race/ethnic strata and the assessment of whether these haplotypes modified the effects of PI exposure on TGs, was performed using the R functions haplo.em and haplo.glm in the haplo.stats library as described by Lake et al. [42]. This approach allows for estimating and testing interaction effects between haplotypes and PI exposure when linkage phase is ambiguous and employs an expectation-maximization type algorithm that iterates between estimating haplotype probabilities and estimating regression parameters.

### Sample Size Considerations

The sample size provides greater than 80% power to detect moderate PI genotype interaction effect sizes of 0.23, 0.36, and 0.35 within Whites, Blacks, and Hispanics, respectively, using a two-sided level = 0.05 test, and within-cell respective assumed standard deviations of 0.7, 0.5, and 0.6 based on observed data (NCSS 2001 PASS 2000.) Notably, our analysis is not able to rule out smaller underlying interaction effects.

## Results

Demographic and clinical information by study are provided in Table S1. Median age of the cohort was 41 (IQR: 36 to 48), 89% were male, and 39 patients were on lipid-lowering therapies. Median CD4 was 442 (IQR: 294.3 to 642.8) and 91.1% of patients had RNA copies <400. The proportion of Black participants (19.3%) was somewhat lower than that enrolled in all ACTG studies (27%) or receiving HIV care (33%) [43], but overall gender, race, and age distributions in our study sample were consistent with population estimates for ART-treated HIV adults in the United States. As expected from ACTG study-specific criteria, a number of characteristics, including type of ART, race/ethnicity, use of lipid-lowering therapy, and lipid levels, differed across studies, underscoring the need to control for potential confounding by study.

Summaries of lipid outcomes by race/ethnicity and gender are presented in Table 1. Black participants tended to have lower TGs and non-HDL-c and higher HDL-c than Whites and Hispanics. As expected, participants on any number of PIs (compared to those not on PIs) tended to have higher TGs and non-HDL-c. Consistent with previous reports [5,15], the most abnormal lipid profiles were observed for participants on RTV-containing PI regimens.

**Table 1:**
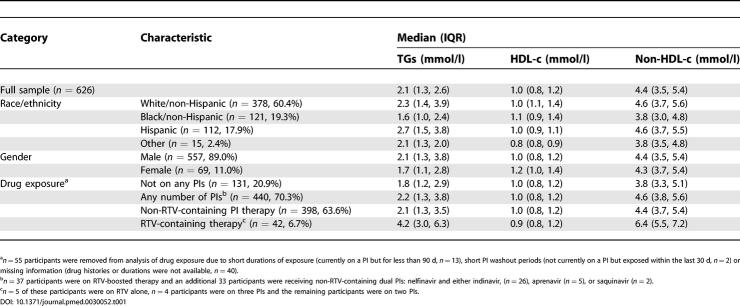
Lipid Profiles by Race/Ethnicity, Gender, and PI Exposure

### Association of Race/Ethnicity, PI Exposure, and ApoC-III/ApoA-I Genotypes with Plasma Lipids

Race/ethnicity was a highly statistically significant predictor of plasma lipids even after controlling for gender, study, age, CD4 count, use of lipid-lowering therapy, and ART drug exposure (Table 2). Overall, Blacks had lower TG and non-HDL-c and higher HDL-c than Whites and Hispanics in fully adjusted models. PI exposure was also a significant predictor of TGs and non-HDL-c with participants receiving RTV-containing PI therapy having an estimated 1.46-fold greater TG levels and 1.25-fold greater non-HDL-c levels compared with participants receiving non-PI therapies. There were no significant associations between individual apoC-III/apoA-I SNPs and plasma lipids in fully adjusted models (Table S2.) Overall, these analyses are limited due to potential for differential PI effects on lipids across racial/ethnicity and the modifying effects of apoC-III/apoA-I genotypes on PI association with lipids.

**Table 2:**
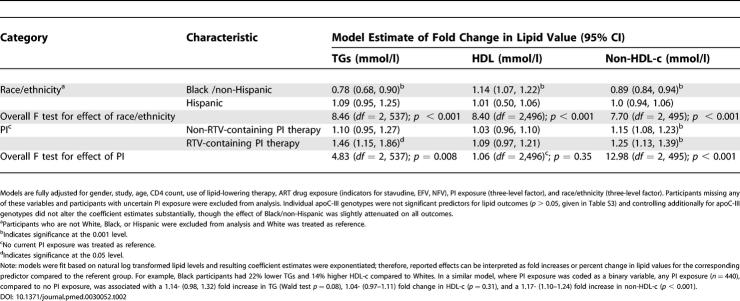
Multivariable Adjusted Association of Race/Ethnicity and PI Exposure with Lipid Outcomes

### Modification of PI Effects on Plasma TG by Race/Ethnicity

**Figure 1:**
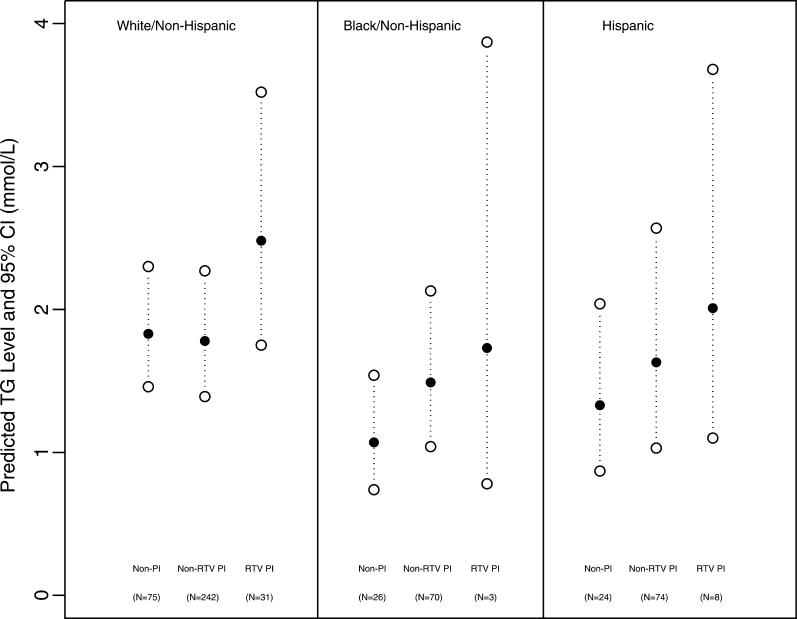
Predicted TG Levels for PI- and Non-PI–Exposed Individuals by Race/Ethnicity Estimated fold increases (95% CI) for non-RTV–containing PI regimen (compared to no current PI exposure) are 0.97 (0.80, 1.18) for White/non-Hispanics, 1.39§ (1.02, 1.89) for Black/non-Hispanics, and 1.23 (0.89, 1.69) for Hispanics. Estimated fold increases (95% CI) for RTV-containing PI regimen (compared to no current PI exposure) are 1.35§ (1.00, 1.83) for White/non-Hispanics, 1.62 (0.75, 3.50) for Black/non-Hispanics, and 1.51 (0.91, 2.52) for Hispanics. § Indicates significantly different than 1.0-fold at the 0.05 level.

As illustrated in Figure 1 **,** current exposure to non-RTV- and RTV-containing PI regimens (compared to no current PI exposure), without consideration for genotypes, tended to be associated with higher TGs, and the strength of this association varied across race/ethnicity. Of note, non-RTV–containing PI regimens were associated with increased plasma TGs in Black/non-Hispanics (estimated fold increase = 1.39, 95% CI = [1.02, 1.89]). This trend was also observed in Hispanics (estimated fold increase = 1.23, 95% CI = [0.89, 1.69]), but not in Whites (estimated fold decrease = 0.97, 95% CI = [0.80, 1.18]). RTV-containing PI therapy appears to be associated with even greater increases in TGs within all racial/ethnic groups, though the within-strata numbers of participants on RTV are small.

### Modification of PI Effects on Plasma TG by ApoC-III/ApoA-I Genotypes within Race/Ethnic Strata

Genotype frequencies varied markedly across race/ethnicity (Table S3). For example, 53.4% of Whites had the apoC-III-482CC genotype, while only 8.3% of Blacks and 35.7% of Hispanics had this genotype. Similar race/ethnicity-specific differences were seen for apoC-III, −455, and apoC-III Intron 1. After stratifying by race/ethnicity, tests of departures from Hardy-Weinberg Equilibrium were not significant for any of the SNPs (unpublished data). The estimated haplotype frequencies (given in Table 3) varied similarly across race/ethnicity with the “wild-type” haplotye (−482C, −455T, Intron 1G, Gly34GlyC, 3238C) frequency estimated at 45.5% in Whites, 10.8% in Blacks, and 35.1% in Hispanics.

**Table 3:**
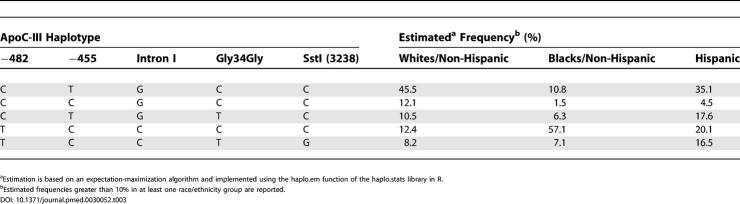
Estimated ApoC-III Haplotype Frequencies by Race/Ethnicity

Table 4 illustrates the interactions between PI exposure and each of the six SNPs in apoC-III/apoA-I on TG levels within race/ethnicity strata. The effect of PI exposure on TG levels varied by apoC-III-482, −455, and intron 1 genotypes in Hispanics. As an illustration, the effect of PI exposure for Hispanic participants with no variant alleles at apoC-II-482 was to increase TGs 1.79-fold, while the effect of PI exposure for Hispanic participants with one or more variant alleles at apoC-III-482 was to decrease TGs 0.97-fold ( = 1.79 × 0.54). Predicted TG levels by race/ethnicity, PI exposure, and genotype are given in Table 5 and provide an illustration of these interaction effects.

**Table 4:**
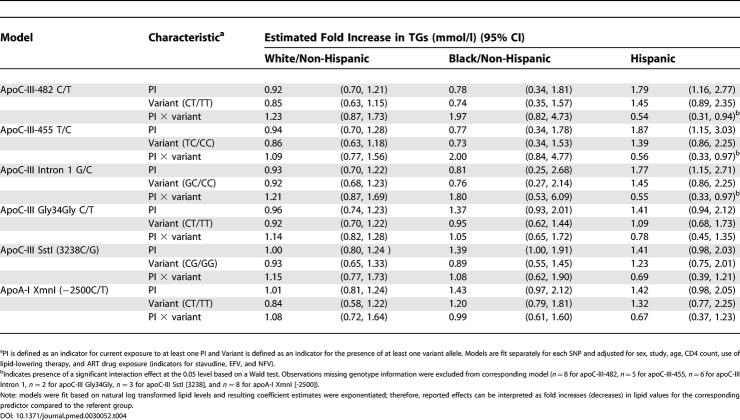
Interaction Effects of Variants in ApoC-III /ApoA-I with PI on Plasma TG by Race/Ethnicity

**Table 5:**
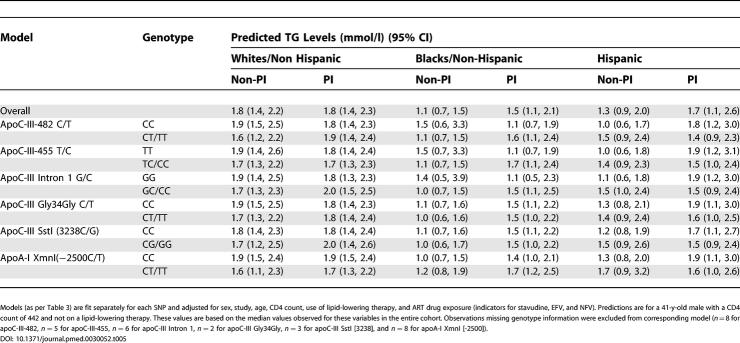
Predicted TG Levels by Genotype, Race/Ethnicity, and PI Exposure

A significant difference in the effect of PI exposure on TGs across genotypes in Whites and in Blacks was not detectable. However, predicted TG levels across genotypes did differ across the three ethnic groups (Table 5), suggesting race-specific differences in the influence of apoC-III genotypes on PI-related dyslipidemia. The difference in predicted TGs between PI-exposed and unexposed Hispanic participants with the apoC-III-482 CC genotype compared to participants with the CT/TT genotype (+ 0.8 mmol/l versus −0.1 mmol/l) appears to be different from that in Whites (−0.1 mmol/l versus + 0.3 mmol/l) and Blacks (−0.4 mmol/l versus + 0.3 mmol/l). Thus, in contrast to Hispanics, PI exposure tended to be associated with higher TGs in Whites and Blacks with the −482 CT/TT genotypes, but not in those with the CC genotype. In fully adjusted models that combined racial/ethnic groups, the three-way interaction effects between PI use, SNPs, and Hispanic race/ethnicity were marginally significant for apoC-III-482 (*p* = 0.07), apoC-III-455 (*p* = 0.08), apoC-III Intron 1 (*p* = 0.03), and apoA-I XmnI (−2500T/C) (*p* = 0.06).

### Modification of PI Effects on Plasma TG by Composite ApoC-III Genotypes within Race/Ethnic Strata

The tests of interaction between each SNP and PI exposure are not independent due to the high degree of concordance across genotypes: *n* = 96 out of 104 (92.3%) Hispanics were concordant in their genotypes for −482 and −455 (i.e., they were wild-type for both or variant for both based on the binary SNP coding); *n* = 99 of 104 (95.2%) were concordant for −482 and Intron 1; and *n* = 92 of 104 (88.5%) were concordant for all three SNPs.

**Figure 2:**
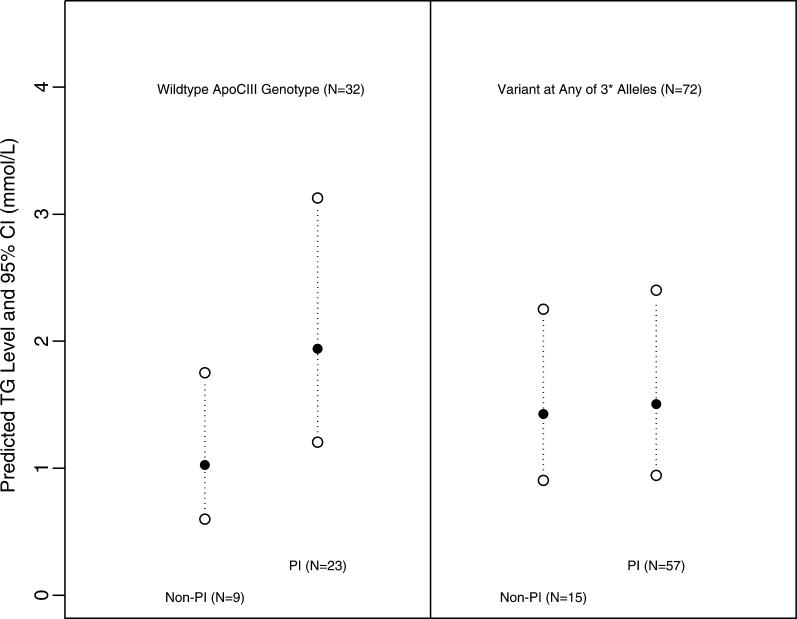
Predicted TG Levels for PI- and Non-PI–Exposed Hispanics by Composite ApoC-III Genotype Variable *ApoCIII-482 (CT/TT), −455 (TC/CC), and/or SstI (3238) CG/GG. The negative effect of PI exposure on TGs is greatly attenuated (56% less) among Hispanics with one or more apoC-III variant compared to Hispanics with the wild-type genotype at these loci (95% CI = [0.32, 0.97], *p*-value = 0.04.)

Consistent with the single SNP analysis, there appears to be a protective effect of the composite variant genotype, (apoC-III −384, −355, and SstI [3238]) on the association of PIs with TG levels within Hispanics (interaction effect = 0.56, 95% CI = [0.32, 0.97], *p*-value = 0.04). Among Hispanic participants with no variant alleles at the three apoC-III SNPs indicated (*n* = 72), PI exposure (*n* = 57) was associated with 1.89-fold higher plasma TG levels compared to the non-PI–exposed group (*n* = 15); however, among Hispanic participants with a variant allele at one or more of the three apoC-III SNPs indicated (*n* = 32), the increase in TGs in PI-exposed participants (*n* = 23) versus non-PI–exposed subjects (*n* = 9) was only 1.06-fold (Figure 2). Similar to the single SNP analysis, the three-way interaction between PI use, genotype, and race/ethnicity was marginally significant (0.055) with an estimated fold decrease of 0.52. Analyses did not reveal significant haplotype PI-interaction effects on TGs. However, within Hispanics, the interaction effects of the non-wild-type haplotypes with PI exposure were 0.84- and 0.75-fold decreases for additive or dominant models respectively, which is consistent with the direction and magnitude of effects observed for composite genotypes and individual SNP analyses.

## Discussion

Our study is the first of race/ethnicity influences on plasma lipoproteins and is the largest study of genetic effects on lipids in HIV-1-infected patients. We found that race/ethnicity was a predictor of plasma lipids in HIV-1 patients on ART. Overall, Black patients on ART had a less atherogenic lipid profile compared to Whites and Hispanics. This finding is consistent with epidemiological data in non-HIV-1 populations [31–33], but may be of particular importance given the worldwide demographics of HIV-1 infection. Despite lower plasma lipids, the effect of PI exposure on TGs was most marked in Blacks, and, in fact, the effect of non-RTV PI therapy appeared to be stronger in both Blacks and Hispanics compared to Whites. We also found a significant difference in the influence of apoC-III genotypes and PI use across ethnic groups in the association with TGs. Hispanic patients with individual apoC-III variant alleles, or across a composite apoC-III genotype, had lower TGs than patients with the wild-type genotypes when on PI therapy. This interaction was not observed in Whites and Blacks. Overall, these results provide evidence for race-specific differences in both the occurrence of dyslipidemia on ART as well as in the influence of genetic factors on the prevalence of PI-related lipid abnormalities.

Linkage and association studies have established a relationship of apoC-III genotypes, and the apoA-I/CIII/AIV/AV gene cluster, with alterations in plasma TGs [23,28,29,39,44,45]. However, little is known of the molecular mechanisms [24]. The concurrence of SNPs in these linked genes complicates the assignment of metabolic effects to individual variants. For example, most [46,47] evidence suggest that two apoC-III promoter SNPs (−482C/T and −455T/C) attenuate the inhibitory action of insulin on apoC-III gene expression and influence plasma lipids [46,48]. However, almost all studies suggest that these SNPs are associated with lipid levels through their LD with the well studied 3′ UTR SstI (3238) variant [29,30,49,50], which may, in turn, be associated with TG levels through LD with variants elsewhere in the cluster [39,45]. In fact, apoA-I/CIII/AIV/AV SNP and haplotype frequencies vary markedly across ethnic groups [36–38], but most studies of the gene cluster have been restricted to Whites. Furthermore, several environmental factors [51–54], including race/ethnicity [38,40], appear to modify the impact of apoC-III variation on lipids and metabolic traits. For example, in a tri-ethnic sample (*n* = 1,366) Waterworth et al. [38] found an association of the −482T allele with increases in metabolic parameters in Whites, but decreases in these readouts in African-ancestry individuals.

Recent work has demonstrated a potential interaction of lipoprotein genes with PI therapy in promoting lipid abnormalities in HIV-1-infected patients. Fauvel et al. studied 60 consecutive, PI-treated (mostly indinavir) White males with HIV-1, who were also taking two nucleoside reverse transcriptase inhibitors, and found that the rare apoC-III −455C, −482T, or SstI-S2(G) variants were associated with higher TGs and apoB lipoproteins as well as lower HDL-c [13]. Tarr et al. assessed the effect of apoE and apoC-III genotypes on change in lipids on ART in HIV-1-infected patients (*n* = 329) [15]. They found that those patients on RTV, who also had apoE variants (non 3/3) and all three −482T, −455C, and 3238G (SstI) apoC-III polymorphisms, had marked increases in TG levels.

Both of these studies, predominantly of Whites, concluded that the apoC-III-482 (CT and TT), −455 (TC and CC), and 3238 (SstI) (GC and GG) genotypes may be associated with increased TG levels in HIV-1-infected patients on PIs. We did not detect significant interaction effects of these apoC-III genotypes with PIs on plasma TGs in Whites. Despite this, our findings are broadly consistent with these papers in that TG levels in White and Blacks with these genotypes on PIs in our study tended to be higher than individuals not on PI therapy; however, the magnitude of effects on lipids were more modest. These differences may reflect study design and confounding variables including age, gender, concurrent use and type of non-PI ART, the prevalence of diabetes, and use of lipid-lowering medications. Furthermore, we did not consider apoE or other candidate genes that might further modify the association of PIs with dyslipidemia. Notably, in Tarr et al. the interaction with PI therapy was only significant in those individuals with the combination of apoE and apoC-III variants [15].

As hypothesized, we found evidence of race/ethnic differences in the association of apoC-III genotypes with PI-related dyslipidemia as well as marked race/ethnicity specific differences in SNP and estimated haplotype frequencies. Although our findings of a potential protective effect of the non-wild-type apoC-III variants on PI-induced hypertriglyceridemia in Hispanics is not consistent with studies in White samples, they are not surprising in the context of prior studies that demonstrated complex transcriptional regulation of the apoA-I/CIII/AIV/AV gene cluster [55], race/ethnic specific LD across this region [36–38], interplay of distinct alleles that confer susceptibility/resistance to hypertriglyceridemia [28,29,39,45,50], and prior evidence for modification of apoC-III effects by race/ethnicity [38,40]. Our finding does not imply that “race” is responsible for specific differences in SNP functional effects. Rather, it is much more likely that differences relate to race/ethnicity-specific LD with unmeasured functional variants in the apoA-I/C-III/AIV/AV cluster, or to the confounding influence of additional environmental or genetic factors which also vary with race/ethnicity. For these reasons, we believe that our findings are not in conflict with published studies including those in HIV-1 samples [13,15].

Race/ethnicity is often considered a surrogate for environmental influences on lipids, but recent studies demonstrate that genetic factors also account for important differences in plasma lipids across ethnic groups [34,56]. For example, hepatic lipase SNPs, present only in African Americans, result in lower enzyme activity and account for a significant proportion of the race/ethnic differences in HDL-c levels [34]. Recently, Cohen et al. reported that loss-of-function mutations in proprotein convertase subtilisin kexin-9, a gene that regulates hepatic low density lipoprotein receptor expression, were associated with markedly lower plasma levels of low density lipoprotein and that these mutations were relatively common in African Americans but rare in European Americans [56]. In this context, it is likely that genetic variation, as well as environmental factors, contribute to significant region-specific differences in lipid and metabolic complications of ART, such as those noted in the recently completed 2NN multi-national clinical trial [57].

The main limitation of our study is that it was cross-sectional, involved a heterogeneous, multi-ethnic sample across ACTG studies, and had limited power to detect small interaction effects within race/ethnic strata. Our goal was to characterize trends in the data, and we caution against placing too much emphasis on the absolute *p*-values, since type I errors due to multiple testing are possible with subgroup analyses. Due to the highly correlated nature of many of the reported tests, induced by the overlap in genotypes across SNPs, we included an analysis of a single composite genotype variable that is consistent with multi-locus analysis approaches in the literature [13,15,58,59]. While this approach is not a correction for multiple testing, it lends strong support to the single SNP analysis and is an appropriate alternative for our setting. Our findings require further evaluation, ideally in large prospective cohort or randomized trial settings that determine changes in lipids over time and also allow a careful assessment of additional environmental (e.g., diet and smoking) and genetic factors (e.g., apoE and lipase genotypes). However, to date this is the largest pharmacogenetic study in HIV-1 to address ethnic variation in plasma lipoproteins in patients on ART.

Overall, our findings of race/ethnicity-specific apoC-III-PI interaction on plasma TGs should be interpreted cautiously. Based on an examination of 43 meta-analyses of genetic association studies, Ioannidis et al. concluded that the biological impact of genetic markers on risk for common diseases is usually consistent across racial boundaries [60]. However, they did find almost a 3-fold greater occurrence of race-specific heterogeneity in genetic effects than would be expected by chance. Thus, the challenge lies in identifying which genetic effects may be influenced by race/ethnicity and what is the basis of such influences. Our findings caution against generalizing results of genetic studies in White samples when considering metabolic traits in HIV-1 populations, particularly for genes with known complex LD structures reported to vary by race/ethnicity.

In summary, this study provides novel information regarding HIV-1 subgroups that may be at differential risk of developing metabolic and CV complications of ART. Approaches that account for racial/ethnic heterogeneity are mandated in pharamcogenetic studies of such complications given the multi-ethnic distribution of HIV-1 infection, concerns regarding future atherosclerotic CV disease in these populations, and the potential to use alternative strategies [16,61] to reverse or avoid metabolic effects of ART.

## Supporting Information

Table S1 Demographic and Clinical Characteristics by Study (134 KB DOC).

Table S2 Individual SNP Effects on Lipids in Multivariable Adjusted Models with Racial/Ethnic Groups Combined (37 KB DOC).

Table S3 Genotype Frequencies by Race/Ethnicity (46 KB DOC).

### Accession Numbers

The EntrezGene (prior LocusLink) accession numbers for genes/proteins referred to in this paper are apoA-I (335), apoA-IV (337), apoA-V (116519), apoC-III (345), and apoE (348).

**Patient Summary**

**Background**

One of the unfortunate side effects of protease inhibitors—one of the types of drugs used in treating HIV—is that they affect lipids in the body; for example, they cause the level of triglycerides to increase in such a way as to make it more likely that patients will develop diseases associated with abnormal lipids such as heart disease and stroke. As these drugs are taken for very long periods of time, there are concerns that there may be a future epidemic of cardiovascular disease in patients with HIV-1 who have taken such lifelong treatment.

**Why Was This Study Done?**

The authors wanted to see if they could identify individuals with HIV who are at increased risk of getting the lipid abnormalities caused by these drugs; in particular, they wanted to see if there were differences across different racial/ethnic groups and according to common variation in some lipid genes. Knowing about such differences might help in deciding between the various different drug treatments available.

**What Did the Researchers Do and Find?**

They looked at 626 people who had taken part in previous studies of HIV drugs, and who had agreed to have their blood stored for further studies such as these. They found that race/ethnicity could help predict levels of plasma lipids, and that the effect of taking protease inhibitors also appeared to differ across race/ethnicity groups. Overall, Black patients taking these drugs were less likely to have the lipid profiles that lead to cardiovascular disease, compared to Whites and Hispanics. However, individuals in all racial/ethnic groups had increases in triglyceride levels when they were given these drugs. Furthermore, Hispanics, but not Whites or Blacks, with variation in the apoC-III gene, appeared to be protected from the triglyceride-raising effect of protease inhibitors

**What Do These Findings Mean?**

Given that HIV is a disease that affects all racial/ethnic groups, it will be important in the future to study carefully and in large numbers the patients of all racial/ethnic groups in studies of metabolic and cardiovascular complications of drugs used to treat HIV. It may also be necessary to look at the effect of certain genes on these lipids. In the future it may be possible to use this information to decide which patients should receive which drugs.

**Where Can I Get More Information Online?**

AIDSinfo is a site run by the National Institutes of Health in the US that has many pages of information on HIV. Searching for “lipid” will bring up fact sheets on HIV drugs and the type of lipid changes discussed in this article:

http://aidsinfo.nih.gov

TheBody.com is an HIV/AIDS resource that can be searched for information on the effects of these drugs on lipids. One page to start at is:

http://www.thebody.com/treat/cardiac.html

## Abbreviations

ACTG: AIDS Clinical Trial Group
apo: apolipoprotein
ART: antiretroviral therapy
CV: cardiovascular
CI: confidence interval
EFV: efavirenz
HDL-c: high-density lipoprotein cholesterol
IQR: interquartile range
LD: linkage disequilibrium
NFV: nelfinavir
PI: protease inhibitor
RTV: ritonavir
SNP: single nucleotide polymorphism
TG: triglyceride
