# Supplementary material for: Associations among Race/Ethnicity, ApoC-III Genotypes, and Lipids in HIV-1-Infected Individuals on Antiretroviral Therapy
Source: PLoS Med. 2006 Jan 24;3(3):e52. doi: 10.1371/journal.pmed.0030052 (PMC1334223; doi:10.1371/journal.pmed.0030052)
Supplement: Supplementary file 1 [file pmed.0030052.st001.doc]

**Appendix Table A: Demographic and Clinical Characteristics by Study**

|  | |  | | | **Overall**  **(N=626)** | | **ACTG372**  **(N=121)** | **A5005s**  **(N=178)** | **A5068**  **(N=69)** | **A5087**  **(N=113)** | **A5116**  **(N=145)** |
| --- | --- | --- | --- | --- | --- | --- | --- | --- | --- | --- | --- |
| **Race** | | | |  | |  |  |  |  |  |  |
|  | White/Non-Hispanic | | | | N=378 | | N=76 | N=101 | N=41 | N=81 | N=79 |
|  |  | | | | 60.4% | | 62.8% | 56.7% | 59.4% | 71.7% | 54.5% |
|  |  | | | |  | | 20.1% | 26.7% | 10.8% | 21.4% | 20.9% |
|  |  | | | |  | |  |  |  |  |  |
|  | Black/Non-Hispanic | | | | N=121 | | N=15 | N=49 | N=19 | N=8 | N=30 |
|  |  | | | | 19.3% | | 12.4% | 27.5% | 27.5% | 7.1% | 20.7% |
|  |  | | | |  | | 12.4% | 40.5% | 15.7% | 6.6% | 24.8% |
|  |  | | | |  | |  |  |  |  |  |
|  | Hispanic | | | | N=112 | | N=25 | N=25 | N=7 | N=22 | N=33 |
|  |  | | | | 17.9% | | 20.7% | 14.0% | 10.1% | 19.5% | 22.8% |
|  |  | | | |  | | 22.3% | 22.3% | 6.3% | 19.6% | 29.5% |
|  |  | | | |  | |  |  |  |  |  |
|  | Other | | | | N=15 | | 5 | 3 | 2 | 2 | 3 |
|  |  | | | | 2.4% | | 4.1% | 1.7% | 2.9% | 1.8% | 2.1% |
|  |  | | | |  | | 33.3% | 20.0% | 13.3% | 13.3% | 20.0% |
|  |  | | | |  | |  |  |  |  |  |
|  |  | | | |  | |  |  |  |  |  |
| **Male** | | | |  | | N=557 | N=105 | N=159 | N=62 | N=103 | N=128 |
|  |  | | | | 89.0% | | 86.6% | 89.3% | 89.9% | 91.2% | 88.2% |
|  |  | | | |  | | 18.9% | 28.5% | 11.1% | 18.5% | 23.0% |
|  |  | | | |  | |  |  |  |  |  |
|  |  | | | |  | |  |  |  |  |  |
| **Age** | | |  | | 41 (36,48) | | 43 (37,48) | 37 (31,44) | 43 (39,49)) | 42 (38,47) | 43 (36,51) |
|  |  | | | |  | |  |  |  |  |  |
| **Current Drug Exposure** | | |  | |  | |  |  |  |  |  |
|  | PI containing regimen | | | | N=440 | | N=109 | N=102 | N=25 | N=88 | N=116 |
|  |  | | | | 70.3% | | 90.1% | 57.3% | 36.2% | 77.9% | 80.0% |
|  |  | | | |  | | 24.8% | 23.2% | 5.7% | 20.0% | 26.4% |
|  |  | | | |  | |  |  |  |  |  |
|  | Non-PI regimen | | | | N=131 | | N=2 | N=66 | N=31 | N=22 | N=10 |
|  |  | | | | 20.9% | | 1.7% | 37.1% | 44.9% | 19.5% | 6.9% |
|  |  | | | |  | | 1.5% | 50.4% | 23.7% | 16.8% | 7.6% |
|  |  | | | |  | |  |  |  |  |  |
|  | D4T containing regimen | | | | N=239 | | N=19 | N=91 | N=27 | N=63 | N=39 |
|  |  | | | | 38.2% | | 15.7% | 51.5% | 39.1% | 55.8% | 26.9% |
|  |  | | | |  | | 7.9% | 38.1% | 11.3% | 26.4% | 16.3% |
|  |  | | | |  | |  |  |  |  |  |
|  | EFV containing regimen | | | | N=264 | | N=2 | N=121 | N=20 | N=55 | N=66 |
|  |  | | | | 42.2% | | 1.7% | 68.0% | 29.0% | 48.7% | 45.5% |
|  |  | | | |  | | 0.8% | 45.8% | 7.6% | 20.8% | 25.0% |
|  |  | | | |  | |  |  |  |  |  |
|  | NVP containing regimen | | | | N=31 | | N=3 | N=0 | N=16 | N=10 | N=2 |
|  |  | | | | 5.0% | | 2.5% |  | 23.2% | 8.8% | 1.4% |
|  |  | | | |  | | 9.7% |  | 51.6% | 32.3% | 6.5% |
|  |  | | | |  | |  |  |  |  |  |
| **Lipid Lowering Drugs** | | |  | | N=39 | | N=3 | N=3 | N=7 | N=0 | N=26 |
|  |  | | | | 6.2% | | 2.5% | 1.7% | 10.1% |  | 17.9% |
|  |  | | | |  | | 7.7% | 7.7% | 17.9% |  | 66.7% |
|  |  | | | |  | |  |  |  |  |  |
|  |  | | | |  | |  |  |  |  |  |
| **Fasting Glucose (N=525)** | | |  | | 5.1 (4.6, 5.5) | | 5.3 (4.9, 5.9) | 5.1 (4.7, 5.4) | 4.8 (4.3, 5.3) | 4.9 (4.6, 5.4) | 5.1 (4.6, 5.4) |
|  |  | | | |  | |  |  |  |  |  |
|  |  | | | |  | |  |  |  |  |  |
| **Body Mass Index (N=524)** | | |  | | 25 (22, 28) | | 25 (22, 27) | 24 (22, 27) | 25 (23, 26) | 26 (23, 29) | 25 (23, 28) |
|  |  | | | |  | |  |  |  |  |  |
|  |  | | | |  | |  |  |  |  |  |
| **Diabetes** | | |  | | N=23 | | N=7 | N=1 | N=3 | N=1 | N=11 |
|  |  | | | | 3.7% | | 5.8% | 0.6% | 4.3% | 0.9% | 7.6% |
|  |  | | | |  | | 30.4% | 4.3% | 13.0% | 4.3% | 47.8% |
|  |  | | | |  | |  |  |  |  |  |
|  |  | | | |  | |  |  |  |  |  |
| **CD4 Count (N=622)** | | |  | | 442 (294, 643) | | 341 (252, 461) | 385 (216, 559) | 734 (637, 890) | 422 (308, 651) | 479 (417, 653) |
|  |  | | | |  | |  |  |  |  |  |
|  |  | | | |  | |  |  |  |  |  |
| **RNA Copies (N=574)** | | |  | | 91.1% | | 90.7% | 85.4% | 98.6% | 85.4% | 98.6% |
| **(Percentage <400 copies)** | | |  | |  | |  |  |  |  |  |
|  | | |  | |  | |  |  |  |  |  |

Column proportions are reported above row proportions or median (interquartile range) is reported. PI = protease inhibitor; D4T = stavudine; EFV = efavirenz; NVP = nevirapine.
