# Supplementary material for: Associations among Race/Ethnicity, ApoC-III Genotypes, and Lipids in HIV-1-Infected Individuals on Antiretroviral Therapy
Source: PLoS Med. 2006 Jan 24;3(3):e52. doi: 10.1371/journal.pmed.0030052 (PMC1334223; doi:10.1371/journal.pmed.0030052)
Supplement: Supplementary file 2 [file pmed.0030052.st002.doc]

**Appendix Table B: Individual SNP Effects on Lipids in Multivariable Adjusted Models with Racial/Ethnic Groups Combined**

|  | | **Model**¶ **Estimate of Effect on Lipid Value (95% Confidence Interval)** | | |
| --- | --- | --- | --- | --- |
| **Genotypes** | | **TGs (mmol/L)** | **HDL-C (mmol/L)** | **Non-HDL (mmol/L)** |
|  | |  |  |  |
|  | ApoC-III -482 CT / TT | 0.99 (0.89, 1.11) | 1.00 (0.95, 1.05) | 0.97 (0.92, 1.02) |
|  |  |  |  |  |
|  | ApoC-III -455 TC / CC | 0.95 (0.86, 1.07) | 1.01 (0.96, 1.07) | 0.96 (0.92, 1.02) |
|  |  |  |  |  |
|  | ApoC-III Intron I CG / GG | 1.02 (0.91, 1.13) | 0.99 (0.94, 1.05) | 0.99 (0.94, 1.04) |
|  |  |  |  |  |
|  | ApoC-III Gly34Gly CT / TT | 0.96 (0.87, 1.07) | 0.99 (0.94, 1.04) | 0.98 (0.94, 1.03) |
|  |  |  |  |  |
|  | ApoC-III SstI (3238) CG / GG | 0.98 (0.87, 1.11) | 0.98 (0.92, 1.04) | 0.95 (0.90, 1.01) |
|  |  |  |  |  |
|  | ApoA-I XmnI (-2500) CT / TT | 0.96 (0.86, 1.07) | 1.00 (0.95, 1.06) | 0.99 (0.94, 1.04) |
|  |  |  |  |  |

¶ Models are fully adjusted for gender, study, age, CD4 count, use of lipid lowering therapy, ART drug exposure (indicators for D4T, EFV, NVP), PI exposure (3-level factor) and race/ethnicity (3-level factor). Subjects missing any of these variables and subjects with uncertain PI exposure were excluded from analysis. SNPs are treated as binary indicators for the presence of one or more variant allele and models are fit separately for each SNP. Observations missing genotype information were excluded from corresponding model (N=8 for ApoC-III -482, N=5 for ApoC-III -455, N=6 for ApoC-III Intron 1, N=2 for ApoC-III Gly34Gly, N=3 for ApoC-III SstI (3238) and N=8 for ApoA-I XmnI (-2500).)
