# Supplementary material for: Associations among Race/Ethnicity, ApoC-III Genotypes, and Lipids in HIV-1-Infected Individuals on Antiretroviral Therapy
Source: PLoS Med. 2006 Jan 24;3(3):e52. doi: 10.1371/journal.pmed.0030052 (PMC1334223; doi:10.1371/journal.pmed.0030052)
Supplement: Supplementary file 3 [file pmed.0030052.st003.doc]

**Appendix Table C: Genotype Frequencies by Race/Ethnicity**

|  | **Number (% within Race/Ethnicity)** | | |
| --- | --- | --- | --- |
|  | **Whites/Non-Hispanic** | **Blacks/Non-Hispanic** | **Hispanic** |
|  | **(N=378)** | **(N=121)** | **(N=112)** |
| **ApoC-III -482 C/T** |  |  |  |
| CC | 202 (53.4%) | 10 (8.3%) | 40 (35.7%) |
| CT | 142 (37.6%) | 61 (50.4%) | 52 (46.4%) |
| TT | 29 (7.7%) | 49 (40.5%) | 18 (16.1%) |
| **ApoC-III -455 T/C** |  |  |  |
| TT | 143 (37.8%) | 10 (8.3%) | 36 (32.1%) |
| TC | 175 (46.3%) | 55 (45.5%) | 50 (44.6%) |
| CC | 56 (14.8%) | 56 (46.3%) | 25 (22.3%) |
| **ApoC-III Intron I G/C** |  |  |  |
| GG | 185 (48.9%) | 5 (4.1%) | 43 (38.4%) |
| CG | 153 (40.5%) | 39 (32.2%) | 46 (41.1%) |
| CC | 34 (9.0%) | 77 (63.6%) | 23 (20.5%) |
| **ApoC-III Gly34Gly C/T** |  |  |  |
| CC | 203 (53.7%) | 65 (53.7%) | 46 (41.1%) |
| CT | 150 (39.7%) | 50 (41.3%) | 47 (42.0%) |
| TT | 23 (6.1%) | 6 (5.0%) | 19 (17.0%) |
| **ApoC-III SstI (3238C/G)** |  |  |  |
| CC | 314 (83.1%) | 88 (72.7%) | 75 (67.0%) |
| CG | 60 (15.9%) | 31 (25.6%) | 32 (28.6%) |
| GG | 3 (0.8%) | 1 (0.8%) | 4 (3.6%) |
| **ApoA-I XmnI (-2500C/T)** |  |  |  |
| CC | 279 (73.8%) | 66 (54.5%) | 75 (67.0%) |
| CT | 85 (22.5%) | 45 (37.2%) | 35 (31.3%) |
| TT | 8 (2.1%) | 8 (6.6%) | 2 (1.8%) |
